# A study update newsletter or Post-it® note did not increase postal questionnaire response rates in a falls prevention trial: an embedded randomised factorial trial

**DOI:** 10.12688/f1000research.14591.2

**Published:** 2019-02-19

**Authors:** Sara Rodgers, Illary Sbizzera, Sarah Cockayne, Caroline Fairhurst, Sarah E. Lamb, Wesley Vernon, Judith Watson, Catherine Hewitt, David Torgerson

**Affiliations:** 1 York Trials Unit, Department of Health Sciences, University of York, York, YO10 5DD, UK; 2 Centre for Rehabilitation Research, Nuffield Department of Orthopaedics, Rheumatology and Musculoskeletal Sciences, University of Oxford, Oxford, UK; 3 Division of Podiatry and Clinical Sciences, School of Human and Health Sciences, University of Huddersfield, Huddersfield, HD1 3DH, UK

**Keywords:** Randomised controlled trial; randomisation; embedded trial; newsletter; Post-it® note; response rate

## Abstract

**Background: **Participants not returning data collection questionnaires is a problem for many randomised controlled trials. The resultant loss of data leads to a reduction in statistical power and can result in bias. The aim of this study was to assess whether the use of a study update newsletter and/or a handwritten or printed Post-it® note sticker increased postal questionnaire response rates for participants of a randomised controlled trial.

**Method: **This study was a factorial trial embedded within a host trial of a falls-prevention intervention among men and women aged ≥65 years under podiatric care. Participants were randomised into one of six groups: newsletter plus handwritten Post-it®; newsletter plus printed Post-it®; newsletter only; handwritten Post-it® only; printed Post-it® only; or no newsletter or Post-it®. The results were combined with those from previous embedded randomised controlled trials in meta-analyses.

**Results: **The overall 12-month response rate was 803/826 (97.2%) (newsletter 95.1%, no newsletter 99.3%, printed Post-it® 97.5%, handwritten Post-it® 97.1%, no Post-it® 97.1%). The study update newsletter had a detrimental effect on response rates (adjusted odds ratio 0.14, 95% CI 0.04 to 0.48, p<0.01) and time to return the questionnaire (adjusted hazard ratio 0.86, 95% CI 0.75 to 0.99, p=0.04). No other statistically significant differences were observed between the intervention groups on response rates, time to response, and the need for a reminder.

**Conclusions:** Post-it® notes have been shown to be ineffective in three embedded trials, whereas the evidence for newsletter reminders is still uncertain.

## Introduction

Postal questionnaires represent a cost-effective and convenient way of collecting participant-reported outcome data in health research, such as in randomised controlled trials (RCTs). However, attrition (i.e. when participants do not return the questionnaires) is a problem for many RCTs. The resultant loss of data leads to a reduction in statistical power and can result in bias ^1^. Although a number of strategies have been found to reduce attrition ^1, 2^, few of these have been evaluated in the context of healthcare RCTs. A recent systematic review highlighted the need for further research into methods of retaining participants in RCTs ^3^.

A Cochrane systematic review ^4^ evaluating 110 different strategies to improve response rates to postal questionnaires in RCTs identified pre-notification as an effective strategy. The odds of response were increased by nearly half when participants were pre-notified of the impending arrival of the questionnaire (odds ratio (OR) 1.45, 95% CI 1.29 to 1.63); however, there was significant heterogeneity among the results of the 47 included trials (p<0.001). Although there have been several studies evaluating different methods of pre-notification (such as letters, postcards or telephone calls) very few of these have been conducted in a healthcare setting. Only one RCT has evaluated the effectiveness of a pre-notification newsletter to increase response rates ^5^. This study found a statistically significant increase in response rates (OR 1.45, 95% CI 1.01 to 2.10) among participants allocated to receive the pre-notification newsletter.

In this trial, we sent participants a study update newsletter shortly before their 12-month questionnaire was due. While this newsletter was not specifically designed to pre-notify participants of the impending arrival of their 12-month questionnaire, it did serve as a reminder about the REFORM study as participants may not otherwise have received any correspondence since the 6 month time point.

The Cochrane review ^4^ also reported that the appearance of the questionnaire (e.g. making questionnaire materials more personal by using handwritten signatures) can affect response rates. For example, the odds of response increased by a quarter when addresses were handwritten compared to using computer-printed labels (OR 1.25, 95% CI 1.08 to 1.45). We are also aware of six studies that evaluated the effectiveness of attaching a Post-it® note to questionnaires to increase response rate ^6– 8^; four of these were undertaken within an academic setting and reported a statistically significant increase (p<0.05) in responses rates when personalised Post-it® notes were used ^3, 6^.

At the York Trials Unit we have a programme of undertaking studies within a trial (SWATs) ^9^ that aim to evaluate simple interventions to increase response rates to postal questionnaires. Newsletters and Post-it® notes are relatively inexpensive, so even a small benefit is likely to be cost-effective. A single embedded trial will often not have the statistical power to detect a modest difference if there truly was one present; therefore, we have a strategy of repeating our SWATs in order to conduct meta-analyses to strengthen the evidence base. With respect to newsletters sent prior to questionnaires, our previous trial showed a small absolute difference in favour of the intervention, which was borderline statistically significant (p=0.05) ^5^, whereas our two previous studies of Post-it® notes ^7, 8^ produced identical, non-statistically significant ORs (0.97) favouring the control group (no Post-it® note).

We conducted a SWAT to evaluate the effectiveness of a study update newsletter and/or applying a handwritten or printed Post-it® note to the questionnaire as a means of increasing response rates to the 12-month follow-up questionnaire sent to participants in the REFORM trial. This paper presents the results of this sub-study. We also present the results of a meta-analysis of the three ‘Post-it® notes’ and two ‘newsletters sent prior to questionnaires’ studies previously undertaken at York Trials Unit to increase questionnaire response rates in RCTs of health treatments.

## Methods

### Ethical approval

This trial was embedded within the National Institute for Health Research Health Technology Assessment (NIHR HTA) programme funded REFORM (REducing Falls with ORthoses and a Multifaceted podiatry intervention) study (registration number ISRCTN68240461; registration date, 1 ^st^ July 2011; http://www.isrctn.com/ISRCTN68240461) ^10^, which aimed to evaluate the clinical and cost effectiveness of a podiatry intervention for the prevention of falls in older people. Ethical approval for the REFORM study and this embedded sub-study was given by National Research Ethics Service East of England – Cambridge East Research Ethics Committee (REC reference 11/EE/0379) and the University of York, Department of Health Sciences Research Governance Committee.

### Participants

Participants in the REFORM study who were due to be sent their 12-month follow-up questionnaire were included in this nested RCT. Participants who had asked to be withdrawn from the REFORM study or who did not wish to receive a questionnaire at this time point were excluded. Supplementary File 1 contains the full trial protocol of the REFORM study.

### Design and randomisation

We undertook a three-by-two SWAT. Participants were allocated to one of six arms using block randomisation with a block size of 18, stratified by REFORM treatment group allocation. An independent data manager who was not involved in the recruitment of participants generated the allocation sequence by computer and allocated participants in a 1:1:1:1:1:1 ratio.

### Interventions

Participants were assigned to one of the following six groups: study update newsletter plus handwritten Post-it® note applied to the questionnaire; newsletter plus printed Post-it®; newsletter only; handwritten Post-it® note only; printed Post-it® note only; or neither newsletter nor Post-it® note. The newsletter contained information regarding trial progress, including the geographical location and number of participants recruited and what happens at the end of the study [ Supplementary File 2]. The newsletter was posted to participants 3 weeks prior to posting the 12-month questionnaire. Those participants randomised to not receive the newsletter were sent this eight weeks after the questionnaire was sent out. The wording on the Post-it® note was “Please take a few minutes to complete this for us. Thank you! Sarah”. (Sarah was the name of the REFORM Trial Manager.) In order to minimise the possibility of heterogeneity, the wording (except for the name), text size and font on the printed Post-it® note was the same as that used for the studies by Tilbrook *et al*. ^7^ and Lewis *et al*. ^8^ and the Post-it® note was placed in the same location, on the top right hand corner of the questionnaire. Two researchers and three trial secretaries wrote the text of the handwritten Post-it® notes and every effort was made to ensure the format of the message was consistent. All participants also received an unconditional £5 note with their final follow up.

### Management of the postal questionnaires

The date participants were sent and returned their postal questionnaires was recorded. All participants who did not return their follow-up questionnaire within 2 weeks were sent up to two standard reminders, 2 weeks apart, by post, text or email according to the participant’s preference, followed by a telephone reminder 1 week later.

### Primary outcome

The primary outcome was questionnaire response rate defined as the proportion of participants that returned their 12-month postal follow-up questionnaire to York Trials Unit.

### Secondary outcomes

The secondary outcomes were: time to response, defined as number of days between the questionnaire being mailed out to a participant and the questionnaire being recorded as returned to York Trials Unit; and the proportion of participants that needed a reminder.

### Statistical analysis

All statistical analyses were conducted in Stata version 14 (StataCorp. 2015. Stata Statistical Software: Release 14. College Station, TX: StataCorp LP) using two-sided tests at the 5% significance level on an intention-to-treat basis. Age at randomisation into the main REFORM trial, gender and main trial allocation are summarised by randomised sub-study group. This factorial trial is reported as recommended by Montgomery *et al*. ^11^ Response rates were calculated for each intervention. All survey responses were included regardless of how long the questionnaire took to be returned. A logistic regression model containing the two interventions (Post-it® note and newsletter), age, gender and REFORM treatment allocation was performed. Adjusted ORs and corresponding 95% confidence intervals (CIs) were obtained from this model. The presence of an interaction between the two interventions was also tested by introducing the interaction term of the interventions into the logistic model.

Time to return the 12-month follow-up questionnaire was calculated as the number of days from the date the questionnaire was sent out, to the date it was returned. Median time to return was calculated for all participants who returned their questionnaire. For the time-to-event analysis, questionnaires that were not returned or returned 6 weeks (42 days) or more after being sent were treated as censored. Time to questionnaire return was plotted for both interventions using Kaplan-Meier survival curves, and the log-rank test was used to compare the randomised groups within each intervention. A Cox proportional hazards regression model containing the two interventions, age, gender and REFORM treatment allocation was performed; adjusted hazard ratios (HR) and corresponding 95% CIs were obtained. The proportion of participants requiring a reminder was analysed using a similarly adjusted logistic model.

An aggregated random effects meta-analysis of this study with the study reported by Mitchell *et al*. ^5^ evaluated the effect of sending a newsletter before receiving the questionnaire to improve response rates. A second aggregated random effects meta-analysis was conducted incorporating the results of this study and those by Tilbrook *et al*. ^7 ^ and Lewis *et al*. ^8^ in order to evaluate the effect of receiving a questionnaire with an attached Post-it® note on response rates. We also performed a GRADE (Grading of Recommendations Assessment, Development and Evaluation) assessment ^12^ to assess the certainty of the recommendations we have made.

Supplementary File 3 contains a completed CONSORT checklist for this study.

## Results

**Figure 1.**
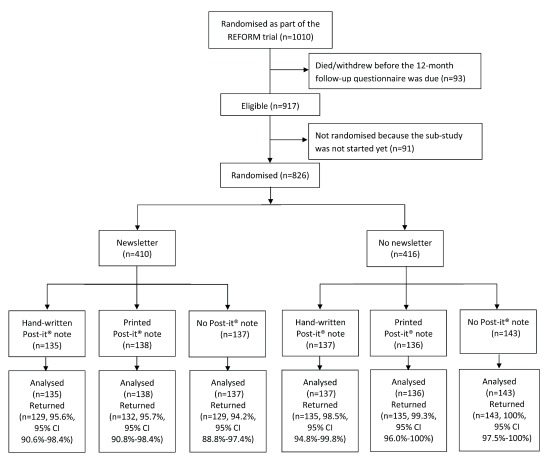
Flow diagram for the REFORM sub-study.

A total of 1010 participants were recruited into the REFORM study and randomised to receive a multifaceted podiatry intervention or usual care. In total, 917 (90.8%) reached the 12-month time point and were sent a follow-up questionnaire, of which 826 (90.1%) were randomised into this embedded RCT (due to a delay in the start of the sub-study): 135 to receive the newsletter and the handwritten Post-it® note; 138 to receive the newsletter and the printed Post-it® note; 137 to receive the newsletter only; 137 to receive the handwritten Post-it® note only; 136 to receive the printed Post-it® note only; and 143 to receive neither the newsletter nor the Post-it® note ( Figure 1). Participants had a mean age of 78 years (range 65 to 96 years), and were predominantly female (n=509, 61.6%). Age and main trial allocation were balanced between the six groups, whereas a small chance imbalance for gender can be seen: the presence of women tended to be higher in the groups receiving the newsletter (65.6% vs 57.7%) and higher in the group receiving the hand-written Post-it® note (66.5%) than the printed (60.2%) or no Post-it® note (58.2%) ( Table 1).

**Table 1.** Baseline characteristics of participants.

Variable	Newsletter and handwritten Post-it® note (n=135)	Newsletter and printed Post-it® note (n=138)	Newsletter only (n=137)	Handwritten Post-it® note only (n=137)	Printed Post-it® note only (n=136)	No newsletter or Post-it® note (n=143)
Age, years						
Mean (SD)	78.0 (7.0)	76.9 (6.9)	79.0 (7.0)	77.6 (7.2)	77.5 (6.9)	76.3 (7.0)
(Min–Max)	(65–95)	(65–95)	(65–96)	(65–96)	(65–93)	(65–89)
Median	78	77	80	78	77	77
Gender, n (%)						
Male	39 (28.9)	48 (34.8)	54 (39.0)	52 (38.0)	61 (44.9)	63 (44.1)
Female	96 (71.1)	90 (65.2)	83 (61.0)	85 (62.0)	75 (55.1)	80 (55.9)
Main trial allocation, n (%)						
Control	71 (52.6)	69 (50.0)	71 (51.8)	72 (52.6)	69 (50.7)	75 (52.4)
Intervention	64 (47.4)	69 (50.0)	66 (48.2)	65 (47.4)	67 (49.3)	68 (47.6)

### Questionnaire response rate

The total number of participants returning the 12-month follow-up questionnaire was 803 of 826 (97.2%), 390 of 410 (95.1%) of those who received the newsletter, and 413 of 416 (99.3%) of those who did not receive it. The difference in response rates between these two groups was statistically significant (crude difference in percentages (CDP) 4.2%, 95% CI 1.9% to 6.4%; crude OR 0.14, 95% CI 0.04 to 0.48, p<0.01; adjusted OR 0.14, 95% CI 0.04 to 0.48, p<0.01) ( Table 2). With respect to the Post-it® note intervention, 272 of 280 (97.1%) participants who received no Post-it® note, 267 of 274 (97.4%) participants who received the printed Post-it® note, and 264 of 272 (97.1%) who received the handwritten Post-it® note returned their questionnaire. The Post-it® note intervention did not show a statistically significant effect on the response rate (printed Post-it® vs no Post-it®: CDP 0.3%, 95% CI -2.4% to 3.0%; crude OR 1.15, 95% CI 0.41 to 3.24, p=0.79; adjusted OR 1.06, 95% CI 0.37 to 3.01, p=0.92; handwritten Post-it® vs no Post-it®: CDP 0.0%, 95% CI -2.9% to 2.7%; crude OR 0.98, 95% CI 0.36 to 2.67, p=0.97; adjusted OR 0.91, 95% CI 0.33 to 2.49, p=0.85). There was no statistically significant interaction between the interventions.

**Table 2.** The effect of the newsletter and Post-it® note interventions on trial outcomes.

	OR/HR	Adjusted statistic (SE)	95% CI	p-value
Questionnaire return (Y/N) ^1^				
Newsletter vs no newsletter	OR	0.14 (0.09)	(0.04, 0.48)	<0.01
Printed Post-it® vs no Post-it®	OR	1.06 (0.56)	(0.37, 3.01)	0.92
Handwritten Post-it® vs no Post-it®	OR	0.91 (0.47)	(0.33, 2.49)	0.85
Time-to-return (days) ^1^				
Newsletter vs no newsletter	HR	0.86 (0.06)	(0.75, 0.99)	0.04
Printed Post-it® vs no Post-it®	HR	0.95 (0.08)	(0.80, 1.13)	0.55
Handwritten Post-it® vs no Post-it®	HR	0.90 (0.08)	(0.76, 1.07)	0.22
Reminder required (Y/N) ^2^				
Newsletter vs no newsletter	OR	1.30 (0.26)	(0.88, 1.91)	0.19
Printed Post-it® vs no Post-it®	OR	1.20 (0.30)	(0.74, 1.94)	0.47
Handwritten Post-it® vs no Post-it®	OR	1.47 (0.35)	(0.92, 2.36)	0.11

^1^Logistic regression; ^2^Cox regression. All models contained both the newsletter and Post-it® note intervention terms and were adjusted for age, gender and main trial allocation. SE, standard error; OR, odds ration; HR, hazard ratio.

### Time to return

**Figure 2.**
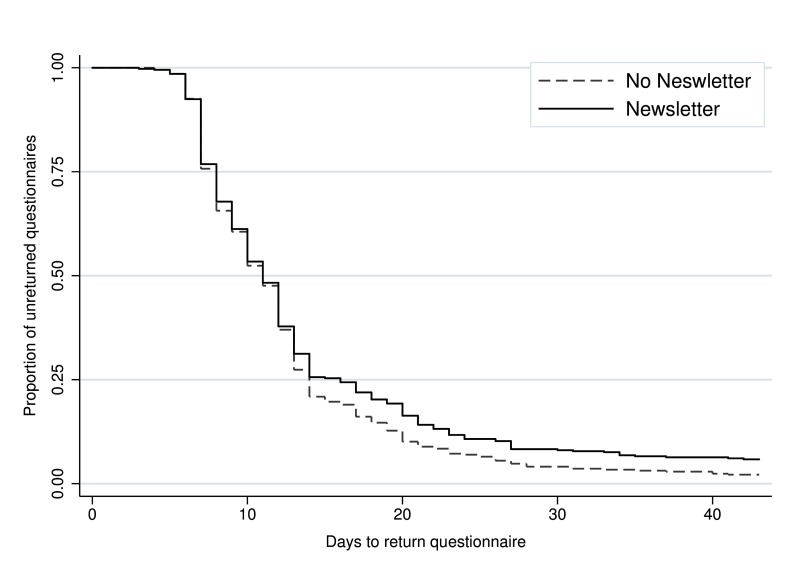
Kaplan-Meier survival curve of time to return for the newsletter intervention.

**Figure 3.**
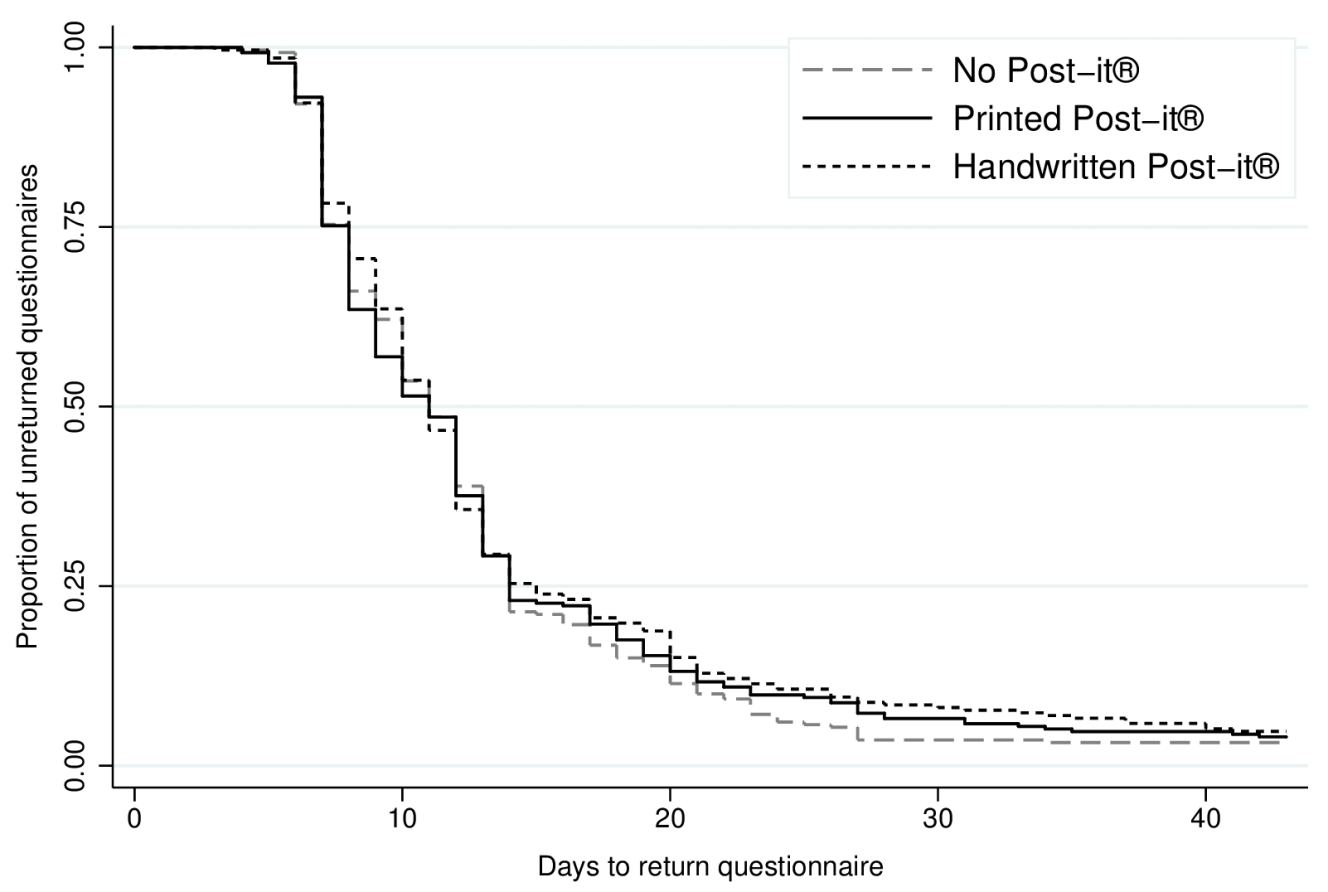
Kaplan-Meier survival curve of time to return for the Post-it® note intervention.

Time to return ranged from 3 to 101 days. Among the participants who responded, the median time taken to return the 12-month questionnaire was 11 days, both overall and in each intervention group (i.e. no newsletter sent, newsletter sent, no Post-it® note, printed Post-it® note, and handwritten Post-it® note). In total, 793 (96.0%) participants returned the questionnaire within 6 weeks (no newsletter: n=407, 97.8%; newsletter: n=386, 94.2%; no Post-it® note: n=271, 96.8%; printed Post-it® note: n=263, 96.0%; and handwritten Post-it® note: n=259, 95.2%). There was evidence of a difference in time to return between those who received the newsletter and those who did not (adjusted HR 0.86, 95% CI 0.75 to 0.99, p=0.04) ( Figure 2; Table 2). The Post-it® note intervention did not appear to have any effect on time to return (printed Post-it® vs no Post-it®: adjusted HR 0.95, 95% CI 0.80 to 1.13, p=0.55; handwritten Post-it® vs no Post-it®: adjusted HR 0.90, 95% CI 0.76 to 1.07, p=0.22) ( Figure 3; Table 2). There was no statistically significant interaction between the interventions.

### Reminders sent

Overall 125 (15.1%) participants required a reminder following 2 weeks of questionnaire non-response (newsletter: n=69, 16.8%; no newsletter: n=56, 13.5%; no Post-it® note: n=36, 12.9%; printed Post-it® note: n=41, 15.0%; handwritten Post-it® note: n=48, 17.7%). There was no evidence of a difference in the proportion of participants requiring a reminder between the groups (newsletter vs no newsletter: adjusted OR 1.30, 95% CI 0.88 to 1.91, p=0.19; printed Post-it® vs no Post-it®: adjusted OR 1.20, 95% CI 0.74 to 1.94, p=0.47; handwritten Post-it® vs no Post-it®: adjusted OR 1.47, 95% CI 0.92 to 2.36, p=0.11) ( Table 2).

### Meta-analysis

**Figure 4.**
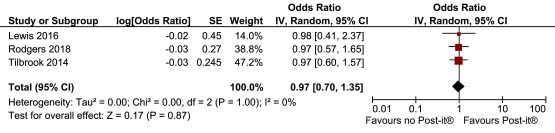
Meta-analyses of Post-it® note interventions.

We combined the two previous Post-it® note studies conducted at York Trials Unit with the study described in this paper. Because there was no material difference in response rates between the printed and handwritten Post-it® note (i.e. 97.5% vs 97.1%) in this study we combined these two groups in the meta-analysis (Post-it® note vs no Post-it® note: adjusted OR 0.98, 95% CI 0.40 to 2.37). The pooled OR was 0.97 (favouring no Post-it® note) but was not statistically significant (95% CI 0.70 to 1.35, p=0.87) ( Figure 4). No heterogeneity was observed (I ^2^=0%). Because no heterogeneity was observed, a sensitivity analysis running a fixed effects meta-analysis was conducted on these data, which produced identical results (to 2 decimal places). As part of the GRADE assessment we assessed the risk of bias of the four trials included in the meta-analyses [ Supplementary File 4]. The GRADE assessment indicated high certainty (i.e. further research is very unlikely to change our confidence in the estimate of effect) [ Supplementary File 5].

**Figure 5.**
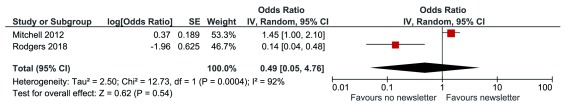
Meta-analysis of newsletters sent prior to questionnaires.

 For the newsletter, the meta-analysis ( Figure 5) showed significant heterogeneity (I ^2^=92%) with a non-statistically significant effect estimate favouring no intervention (pooled OR 0.49, 95% CI 0.05 to 4.76, p=0.54). The GRADE assessment indicated very low certainty (i.e. any estimate of effect is very uncertain) [ Supplementary File 6] largely due to inconsistency between the results of the two studies and imprecision of the estimates.

Raw data concerning patient demographics, type of reminder received and the returning of the questionnaire ^13^ Click here for additional data file. Copyright: © 2019 Rodgers S et al. 2019 Data associated with the article are available under the terms of the Creative Commons Zero "No rights reserved" data waiver (CC0 1.0 Public domain dedication).

## Discussion

We undertook a three-by-two factorial randomised SWAT of a study update newsletter and/or attaching Post-it® notes (printed or handwritten) to postal questionnaires to improve response rates. The trial was embedded at the final (12-month) follow-up time point of the NIHR HTA-funded REFORM RCT. There was evidence that sending a study newsletter 3 weeks prior to the 12-month questionnaire had a detrimental effect on the response rate (adjusted OR 0.14, 95% CI 0.04 to 0.48, p<0.01) and time to return the questionnaire (adjusted HR 0.86, 95% CI 0.75 to 0.99, p=0.04); however, the raw difference in response rates was small (95.1% vs 99.3%). It is possible that the language used in the study update newsletter could have contributed to this as it was not specifically pertaining to pre-notification of the 12-month questionnaire. Instead, the newsletter was initially intended to be sent with the 12-month questionnaire as an acknowledgment of the end of the participant’s involvement in the trial. It therefore indicated that the participant did not need to return any further data relating to falls they experienced. When it was decided to implement this SWAT, the same newsletter was used but was sent 3 weeks prior to the due date of the 12-month questionnaire. In hindsight, the wording of the newsletter may have led participants to believe that they did not need to return the 12-month questionnaire; this may account for its detrimental effect in this trial. A small imbalance in gender among the six groups was observed at randomisation, but gender was adjusted for in all analyses. A previous SWAT of a pre-notification newsletter ^5^, conducted in an older female population, showed a positive finding, which was in line with the Cochrane review ^4^ of pre-notification approaches to enhance survey returns. A meta-analysis combining that trial with ours produced a small, non-statistically significant effect favouring use of a newsletter; however, there was significant heterogeneity in the results and the GRADE assessment we conducted indicates that the level of certainty for this estimate of effect is very low.

Response rates across the groups receiving a printed Post-it® note on their questionnaire, a handwritten Post-it® note and no Post-it® note were all very similar (97.5, 97.1 and 97.1%, respectively). There was no statistically significant difference between the groups in terms of response rate, time to return the questionnaire, and requiring a reminder. This lack of effect on response rates has now been demonstrated across three separate trials. The first trial was among patients with neck pain (mean age, 53 years) ^7^, the second trial was among older patients (mean age, 74 years) at risk of depression ^8^ with the current trial among a similar age group (mean age, 76 years), but no risk/diagnosis of depression. The consistent results suggest that it is not worthwhile undertaking further trials of this intervention among a middle-aged or older population. This is supported by the GRADE assessment which indicates the high certainty of this outcome. There may be merit, however, in testing this intervention in a younger population where response rates may be lower.

No statistically significant differences were observed in the proportion of participants requiring a reminder between the groups.

Response rates in the six groups all exceeded 94%, making significant improvement difficult. These simple interventions were relatively inexpensive but not cost-free due to the price of printing the newsletters and the printed Post-it® notes, and staff time to handwrite the Post-it® notes. A cost-effectiveness analysis was not performed since a benefit was not observed.

## Conclusions

In summary, we found no evidence of a benefit of handwritten or printed Post-it® notes on questionnaire response rates. We also found a negative effect of the study update newsletter; however, a meta-analysis suggests the evidence is still uncertain.

## Data availability

The data referenced by this article are under copyright with the following copyright statement: Copyright: © 2019 Rodgers S et al.

Data associated with the article are available under the terms of the Creative Commons Zero "No rights reserved" data waiver (CC0 1.0 Public domain dedication). 

**Dataset 1. Raw data concerning patient demographics, type of reminder received and the returning of the questionnaire.** DOI: 10.5256/f1000research.14591.d202910 ^13^

## Supplementary material

**Supplementary File 1. Complete trial protocol for the REFORM study.**

**Supplementary File 2. REFORM 12 month newsletter.**

**Supplementary File 3. Completed CONSORT checklist.**

**Supplementary File 4: Assessment of Risk of Bias.**

**Supplementary File 5: GRADE assessment – Post-it Notes.**

**Supplementary File 6: GRADE assessment – Newsletters.**
