# Supplementary material for: A study update newsletter or Post-it® note did not increase postal questionnaire response rates in a falls prevention trial: an embedded randomised factorial trial
Source: F1000Res. 2019 Feb 19;7:1083. Originally published 2018 Jul 16. [Version 2] doi: 10.12688/f1000research.14591.2 (PMC6402081; doi:10.12688/f1000research.14591.2)
Supplement: Supplementary file 3 [file f1000research-7-19852-s0002.tgz › d60a05cf-f482-4939-ba1a-7b960b9a9a52_Supplementary_File_2._REFORM_12_month_newsletter.pdf]

REFORM

Second Study Update

A big thank you from the REFORM research team for taking part in this study.

Over 1,700 people from nine areas across England and Ireland have taken part in the REFORM trial. This will help us to find out whether a package of podiatry care can help to reduce falls in people over the age of 65.

Over 1700 people recruited to the study!

What happens next?

We have now finished collecting information for the study, so you no longer need to let us know whether you have fallen. If you have any remaining falls calendars that you have not sent to us please dispose of these.

We will analyse the data and write the report. The report will be ready in Summer 2016. We will send you a leaflet to let you know the results of the study. We will also publish the results in journals, present them at conferences and let podiatrists know the results.

If you have given us permission we may contact you to find out if you wish to take part in further related studies. Your contact details will not be shared with anyone else.

Should you need to contact us, here are the details:

Study co-ordinator
XXXXXXXX
Tel: XXXXXXXX or Freephone **XXXXXXXX**
Email: XXXXXXXX
Answer machine available 24 hours a day

Thank you for your help!
