# Supplementary material for: A study update newsletter or Post-it® note did not increase postal questionnaire response rates in a falls prevention trial: an embedded randomised factorial trial
Source: F1000Res. 2019 Feb 19;7:1083. Originally published 2018 Jul 16. [Version 2] doi: 10.12688/f1000research.14591.2 (PMC6402081; doi:10.12688/f1000research.14591.2)
Supplement: Supplementary file 5 [file f1000research-7-19852-s0004.tgz › 6455416f-b584-4e3c-8b39-9a2e1d7d870a_Supplementary_File_4._Assessment_of_Risk_of_Bias.docx]

**Cochrane Risk of bias tool for randomised controlled trials** (Ref: Higgins JPT, Altman DG, Gøtzsche PC, Jüni P, Moher D, Oxman AD, et al. The Cochrane Collaboration’s tool for assessing risk of bias in randomised trials. BMJ. 2011;343:d5928.)

|  | Lewis 2017 | Rodgers 2018 | Tilbrook 2014 | Mitchell 2012 |
| --- | --- | --- | --- | --- |
| Random Sequence Generation | Low risk | Low risk | Low risk | Low risk |
| Allocation Concealment | Low risk | Low risk | Low risk | Low risk |
| Selective Reporting | Unclear | Unclear | Unclear | Unclear |
| Blinding of Participants and Personnel | Low risk | Low risk | Low risk | Low risk |
| Blinding of Outcome Assessment | Unclear | Unclear | Unclear | Unclear |
| Incomplete Outcome Data | Low risk | Low risk | Low risk | Low risk |
| Other | Low risk | Low risk | Low risk | Low risk |
