# Supplementary material for: A study update newsletter or Post-it® note did not increase postal questionnaire response rates in a falls prevention trial: an embedded randomised factorial trial
Source: F1000Res. 2019 Feb 19;7:1083. Originally published 2018 Jul 16. [Version 2] doi: 10.12688/f1000research.14591.2 (PMC6402081; doi:10.12688/f1000research.14591.2)
Supplement: Supplementary file 6 [file f1000research-7-19852-s0005.tgz › c45d20c5-0b0e-4fd9-a698-57cb9f1ac32c_Supplemantary_File_5._GRADE_assessment_-_Post-it_Notes.docx]

**Question**: Post it notes compared to no Post it notes for improving response rates to questionnaires

| **Certainty assessment** | | | | | | | **№ of patients** | | **Effect** | | **Certainty** | **Importance** |
| --- | --- | --- | --- | --- | --- | --- | --- | --- | --- | --- | --- | --- |
| **№ of studies** | **Study design** | **Risk of bias** | **Inconsistency** | **Indirectness** | **Imprecision** | **Other considerations** | **Post it notes** | **no Post it notes** | **Relative (95% CI)** | **Absolute (95% CI)** |  |  |
| **New outcome** | | | | | | | | | | | | |
| 3 | randomised trials | not serious | not serious | not serious | not serious | none | 1069/1906 (56.1%) | 837/1906 (43.9%) | **OR 0.97** (0.70 to 1.35) | **7 fewer per 1,000** (from 85 fewer to 75 more) | ⨁⨁⨁⨁ HIGH |  |

**CI:** Confidence interval; **OR:** Odds ratio
