# Supplementary material for: A study update newsletter or Post-it® note did not increase postal questionnaire response rates in a falls prevention trial: an embedded randomised factorial trial
Source: F1000Res. 2019 Feb 19;7:1083. Originally published 2018 Jul 16. [Version 2] doi: 10.12688/f1000research.14591.2 (PMC6402081; doi:10.12688/f1000research.14591.2)
Supplement: Supplementary file 7 [file f1000research-7-19852-s0006.tgz › 2bc04761-60e2-44bc-89ba-de60ae8cf041_Supplementary_File_6._GRADE_assessment_-_Newsletters.docx]

**Question**: Newsletters compared to no newsletters for improving response rates to questionnaires

| **Certainty assessment** | | | | | | | **№ of patients** | | **Effect** | | **Certainty** | **Importance** |
| --- | --- | --- | --- | --- | --- | --- | --- | --- | --- | --- | --- | --- |
| **№ of studies** | **Study design** | **Risk of bias** | **Inconsistency** | **Indirectness** | **Imprecision** | **Other considerations** | **newsletters** | **no newsletters** | **Relative (95% CI)** | **Absolute (95% CI)** |  |  |
| **New outcome** | | | | | | | | | | | | |
| 2 | randomised trials | not serious | serious | serious | serious | none | 1752/3512 (49.9%) | 1760/3512 (50.1%) | **OR 1.19** (0.84 to 1.70) | **43 more per 1,000** (from 43 fewer to 130 more) | ⨁◯◯◯ VERY LOW |  |

**CI:** Confidence interval; **OR:** Odds ratio
