# Supplementary material for: A study update newsletter or Post-it® note did not increase postal questionnaire response rates in a falls prevention trial: an embedded randomised factorial trial
Source: F1000Res. 2019 Feb 19;7:1083. Originally published 2018 Jul 16. [Version 2] doi: 10.12688/f1000research.14591.2 (PMC6402081; doi:10.12688/f1000research.14591.2)
Supplement: Raw data concerning patient demographics, type of reminder received and the returning of the questionnaire 13 [file f1000research-7-19852-s0000.tgz › fa8f7f8e-6b74-4934-b7b2-c45605461060_Data.zip!/Data/Dataset_description.docx]

**Dataset description**

The dataset contains all the variables needed to reproduce the results presented in the paper.

partid: it is the ID used to uniquely identify each participant

sub_alloc: it indicates which of the six groups the participant was randomised to

letter: it indicates whether the participant was randomised to receive the pre-notification letter or

not. This variable was obtained from sub_alloc.

postit: it indicates whether the participant was randomised to receive a hand-written post-it, a

printed post-it or no post-it at all. This variable was obtained from sub_alloc.

alloc: it indicates the allocation of the participant in the main trial.

age: it is the age of the participant as collected at baseline.

gender: gender of the participant.

datesent: it is the date the questionnaire was sent.

datereturn: it is the date the questionnaire was received by the York Trial Unit.

returned: it indicates whether the questionnaire was returned or not.

reminder: it indicates whether at least one reminder was sent.
