# Supplementary material for: A study update newsletter or Post-it® note did not increase postal questionnaire response rates in a falls prevention trial: an embedded randomised factorial trial
Source: F1000Res. 2019 Feb 19;7:1083. Originally published 2018 Jul 16. [Version 2] doi: 10.12688/f1000research.14591.2 (PMC6402081; doi:10.12688/f1000research.14591.2)
Supplement: Peer review history

# Peer review: Reviewer response for version 2

**DOI:** 10.5256/f1000research.19852.r44676

**Published:** 2019-03-01

**Authors:** Shaun P. Treweek

**Affiliations:** 1 Health Services Research Unit, Institute of Applied Health Sciences, School of Medicine, Medical Sciences and Nutrition, University of Aberdeen, Aberdeen, UK

The authors have made a good job of addressing my suggestions and I have no further comments.

I have read this submission. I believe that I have an appropriate level of expertise to confirm that it is of an acceptable scientific standard.

# Peer review: Reviewer response for version 2

**DOI:** 10.5256/f1000research.19852.r44677

**Published:** 2019-02-20

**Authors:** Phil Edwards, Benjamin Woolf

**Affiliations:** 1 Department of Population Health, London School of Hygiene & Tropical Medicine, London, UK; 2 London School of Hygiene & Tropical Medicine, London, UK

The revised manuscript takes account of the concerns we raised in our initial review.

 Phil Edwards and Benjamin Woolf, London School of Hygiene & Tropical medicine.

We have read this submission. We believe that we have an appropriate level of expertise to confirm that it is of an acceptable scientific standard.

# Peer review: Reviewer response for version 1

**DOI:** 10.5256/f1000research.15878.r40423

**Published:** 2018-12-10

**Authors:** Phil Edwards, Benjamin Woolf

**Affiliations:** 1 Department of Population Health, London School of Hygiene & Tropical Medicine, London, UK; 2 London School of Hygiene & Tropical Medicine, London, UK

**Comments on the manuscript:** 

 Thank you for inviting us to review this manuscript. The authors should be commended for nesting this factorial study within the REFORM study, to provide further evidence on methods to increase response to postal questionnaires. It is vital that researchers conduct this type of nested trial for evidence-based methods research to progress in a cost-effective manner. 

 Overall, we found this to be a well-designed, and well-conducted study. We have a few comments regarding the reporting of the results.

**Title:  **We don’t think this is currently the best wording for the title - the “nested RCT” was not the intervention.

**ABSTRACT** 

 Conclusions:

- The authors say that the evidence for “newsletter reminders” is still uncertain, but we think that the authors meant to say “prenotification newsletters”.  

 

**METHODS ** 

 Interventions:

 The authors could make it clearer to what extent the newsletter was a prenotification intervention; for example, was there a letter with the newsletter explaining that the questionnaire was imminent? Or did text within the Newsletter explain that a questionnaire was imminent? It is currently unclear the extent to which the newsletter warned of the imminent questionnaire, and whether it tried to encourage participants to complete it and return it. Perhaps the Newsletter might be included in the Supplementary material?

**RESULTS** 

 Meta-analyses:

 In the meta-analysis of the Post-it note interventions (figure 4), there is no evidence of heterogeneity among the studies (I-squared=0%) so a **fixed effect** model, rather than a random effect model, is appropriate. We expect that the 95% confidence interval will be narrower around the estimated odds ratio of 0.97, consistent with the conclusion that the study found no evidence of a benefit of the Post-it notes on increasing response.

 In the meta-analysis of prenotification by newsletter interventions (figure 5), there is substantial heterogeneity among the studies (I-squared=92%), so in this case, a **random** **effect** model, rather than a fixed effect model is appropriate. We expect that the 95% confidence interval will be wider around the estimated odds ratio of 1.19, consistent with the conclusion that the magnitude of the effect on response of prenotification by newsletter remains uncertain, but that a moderate effect (e.g. OR=1.5) is still plausible. 

**CONCLUSIONS** 

 We were not persuaded that a study with 826 participants is necessarily “a reasonably sized trial”.

 A study powered to detect an intervention effect equivalent to an odds ratio of OR=1.5 from a baseline response proportion of 97% would require 3,826 participants in each arm (80% power), or 5,121 participants in each arm (90% power). However, a statistically significant reduction in response with the prenotification newsletter was observed with the study sample of 826 participants, and so this may stand as the study result without any need for the authors to comment on the size of the sample.

 In our most recent update to the Cochrane Review (cited in the manuscript), Forty-seven trials (79,651 participants) evaluated the effect on response of contacting participants before sending questionnaires. The odds of response were increased by a half when participants were pre-notified (OR 1.45; 95% CI 1.29 to 1.63). However, there was significant heterogeneity among the trial results (P < 0.00001).  We have recently updated this meta-analysis, for an MSc Epidemiology dissertation (Woolf, B. 2018, unpublished data). In this update, 103 trials were included. Overall, pre-notification increased response 1.38 (95%CI: 1.27-1.49) (pooled result from a random effect model). However, when studies at high or unclear risk of bias were excluded the 95% confidence interval, for the pooled effect of the remaining eight studies, crossed the null. The meta-analysis also found several factors which explained some of the heterogeneity (e.g., the method of pre-notification, using a different method of delivering the pre-notification than the questionnaire, and the risk of bias of the included studies). However, heterogeneity was still present after accounting for these factors.

 Given that the method of pre-notification appears to explain study differences, and that this study is only the second to explore the use of Newsletters as a type of pre-notification, it provides important evidence for further understanding this method for potentially reducing questionnaire non-response. 

**Is the work clearly and accurately presented and does it cite the current literature?** 

 Yes, however, there are some minor modifications which would make the paper easier to read. For example, the presentation of the experimental conditions, although presented accurately, was not intuitively easy to grasp. We personally find it easier to understand factorial randomisation (especially when more complex than 2x2) if a matrix or decision tree is provided, for example, the one shown below.

              Condition

           ________|_________

          |                                  |

      Newsletter           no Newsletter

   _________|________   __|_________   

   |                 |         |          |            |         |

 hand     printed    no    hand      printed   no    

 written  postit    Postit  written  Postit    Postit            

 Postit                             Postit

**Is the study design appropriate and is the work technically sound?**

 Yes

**Are sufficient details of methods and analysis provided to allow replication by others?**

 Yes. However, it would be useful if the length of time period for which a survey response would be included in the study was stated explicitly. *Prima facie*, varying the amount of time participants have for their response to be included in the study could change the results of any potential replication.

**If applicable, is the statistical analysis and its interpretation appropriate?**

 No, we believe that a random effect model, rather than a fixed effect model is appropriate in the meta-analysis of pre-notification by newsletter interventions.

 In addition, we would prefer to see more detail about the adjustments which were made for the main analysis. The authors present an odds ratio adjusted for gender. The adjustment was made because of a baseline imbalance after randomisation. However, it is unclear if the decision to make this adjustment was post hoc or part of a decision procedure in a pre-specified protocol. With this in mind, it would be useful if the authors presented 95% confidence intervals for the crude proportions of responses in each conditions to aid integration of these results, as well as the crude odds ratio.

**Are all the source data underlying the results available to ensure full reproducibility?**

 Yes. However, we believe that the study authors should be more explicit about the source and selection methods of studies included in the meta-analyses. 

**Are the conclusions drawn adequately supported by the results?**

 See our comments above

We have read this submission. We believe that we have an appropriate level of expertise to confirm that it is of an acceptable scientific standard, however we have significant reservations, as outlined above.

# Response

**Published:** 2019-02-07

**Authors:** Sara Rodgers

**Affiliations:** University of York, UK

Points addressed in turn:

 Thank you for your comments.  We have addressed each comment in turn below.  Additionally, the paper has been revised with minor amendments to improve clarity and general readability.

 Thank you, we have suggested the alternative title of “A study update newsletter or Post-it® note did not increase postal questionnaire response rates in a falls prevention trial: an embedded randomised factorial trial”

 Apologies, we did not intend to use the word “pre-notification” to suggest that specific text was included to remind participants of the impending arrival of their 12-month questionnaire. More accurately, we sent a ‘study update’ newsletter to participants, which we have now included in supplementary material. We have removed the use of the term “pre-notification” where necessary and clarified throughout the paper.  

 We have now clarified the content of the newsletter and have added this as supplementary material.

 Thank you, the text and analyses have been amended as appropriate so that both are a random effects meta-analysis. We have also introduced the fixed effects meta-analysis as a sensitivity analysis given the lack of heterogeneity in the Post-it analysis.

 Updated forest plot for newsletter random effect meta-analysis (Figure 5).

 Thank you, we acknowledge the reviewer’s point and have removed the text: “in this reasonably sized trial of 826 participants,”.

 We have revised the CONSORT diagram according to the reviewer’s suggestion.

 All survey responses were included regardless of when they were returned.  Text has been added to the manuscript to clarify this.

 The text and analyses have been amended as appropriate so that both are a random effects meta-analysis, and the forest plot for the newsletter meta-analysis (Figure 5) has been updated.

   

 The decision to adjust for gender was made prior to analysis and was not based on the chance imbalance observed in gender.  The unadjusted odds ratios have been added to the manuscript, and the 95% CI for the proportions have been added to the CONSORT diagram.

 We have clarified that we meta-analysed the similar studies undertaken at York Trials Unit previously.

# Peer review: Reviewer response for version 1

**DOI:** 10.5256/f1000research.15878.r36069

**Published:** 2018-08-03

**Authors:** Shaun P. Treweek

**Affiliations:** 1 Health Services Research Unit, Institute of Applied Health Sciences, School of Medicine, Medical Sciences and Nutrition, University of Aberdeen, Aberdeen, UK

This is a well-written article that was great to read. I particularly liked the recommendations in Discussion re: whether it was worth doing more SWAT evaluations. It would be good to see that sort of clarity more often.

 I only have a few comments, which are listed below.

**Interventions**

 Would it be possible to see the newsletter, or at least the bit relevant to this study? We have the Post-It text but not the newsletters.

**Management of postal questionnaires**

 I’m guessing that the content of the reminders was the same for all participants regardless of which arm they were allocated to but could you confirm this?

**Table 1**

 The gender imbalance does look odd to me. I know that you say that it is due to chance and that is of course plausible but it differs by up to about 15% across the interventions for women and by up to about 15% for men. These percentages amount to 10 - 20 or so individuals in a total sample size of 135-143. I’m not sure that size of difference would come about just by chance although, of course, it could. Are you sure it’s just chance, or a feature of the randomisation/blocking/something else?  

**Table 2**

 Could you consider giving absolute differences for the primary outcome as well as the OR? ORs are always a bit tricky to interpret. 

**Figures 4, 5 and linked text**

 Two points. I think it would be good to do two GRADE assessments of the evidence included in the two forest plots. This isn’t as hard as it sounds. Depending on the design quality of the included studies (and the current 2018 one is good) my guess is that if the two other studies in Fig 4 are good studies, GRADE is high and for Fig 5 it’s moderate because of inconsistency, though you might pull it down for imprecision too (1.19 with a CI of 0.84 - 1.70 seems pretty wide to me). Regardless, I do think it would be good to say something about the certainty of the body of evidence and then link that to your recommendations in the Discussion.

 The second point is that I wasn’t sure why you used a fixed effect model for Fig 5 and a random for Fig 4. My guess is that the random effects model is the one to go for (I’d be surprised if the only differences between studies is random error but that interventions, patients, context etc are at play too). Worth thinking about anyway, especially whether the intention was for the two forest plots to use different models.

I have read this submission. I believe that I have an appropriate level of expertise to confirm that it is of an acceptable scientific standard, however I have significant reservations, as outlined above.

# Response

**Published:** 2019-02-07

**Authors:** Sara Rodgers

**Affiliations:** University of York, UK

Thank you for your comments, which we address in turn below.

 We have now included the study update newsletter in supplementary material.

 This is correct, the reminders were standardised. We have clarified this in the text.

 We noticed that there was a mistake in the following sentence: ‘...the presence of women tended to be higher in the groups not receiving the newsletter (65.6% vs 57.7%)’ – the word ‘not’ has been removed.

 We can think of no other explanation than chance for the imbalance in gender between the groups. 

 These are now included in the manuscript.

 Thank you for your suggestion. We have completed the GRADE assessments and included them in the text. We have also included the assessment tables as Supplementary materials.

 The text and analyses have been amended as appropriate so that both are a random effects meta-analysis, but we have introduced the fixed effects meta-analysis as a sensitivity analysis for the Post-it® notes given the lack of heterogeneity.
